# Clinical and Molecular Characteristics of Rare Malignant Tumors of Colon and Rectum

**DOI:** 10.3390/biology11020267

**Published:** 2022-02-08

**Authors:** Alessandro Ottaiano, Mariachiara Santorsola, Francesco Perri, Ugo Pace, Bruno Marra, Marco Correra, Francesco Sabbatino, Marco Cascella, Nadia Petrillo, Monica Ianniello, Marika Casillo, Gabriella Misso, Paolo Delrio, Michele Caraglia, Guglielmo Nasti

**Affiliations:** 1 Istituto Nazionale Tumori di Napoli, IRCCS “G. Pascale”, Via M. Semmola, 80131 Naples, Italy; mariachiara.santorsola@istitutotumori.na.it (M.S.); f.perri@istitutotumori.na.it (F.P.); u.pace@istitutotumori.na.it (U.P.); b.marra@istitutotumori.na.it (B.M.); m.correra@istitutotumori.na.it (M.C.); m.cascella@istitutotumori.na.it (M.C.); p.delrio@istitutotumori.na.it (P.D.); g.nasti@istitutotumori.na.it (G.N.); 2 Oncology Unit, San Giovanni di Dio e Ruggi D’Aragona University Hospital, Universisty of Salerno, 84131 Salerno, Italy; fsabbatino@unisa.it; 3 AMES, Centro Polidiagnostico Strumentale srl, 80013 Naples, Italy; nadia.petrillo@centroames.it (N.P.); monica.ianniello@centroames.it (M.I.); marika.casillo@centroames.it (M.C.); 4 Department of Precision Medicine, University of Campania “L. Vanvitelli”, Via de Crecchio 7, 80138 Naples, Italy; gabriella.misso@unicampania.it (G.M.); michele.caraglia@unicampania.it (M.C.)

**Keywords:** colon, rectum, rare tumors, genetics, NGS

## Abstract

**Simple Summary:**

Tumors of colon and rectum other than adenocarcinomas represent a neglected issue from clinical and laboratory points of view because of their rarity. In this review, we summarize and describe the rare histologic entities occurring in colon and rectum. Clinical and pathologic characteristics, prognostic behavior, treatments, and altered genes are reported to provide readers with a paramount and comparative perspective. In relation to this, we propose that improvements in registries and multidisciplinary research are warranted to ameliorate their management.

**Abstract:**

The most frequent form of colorectal cancer is represented by adenocarcinoma being about 98% of tumor histological types. However, other rare histotypes can be found in colon and rectum (adenosquamous, goblet cell adenocarcinoma, lymphoma, medullary carcinoma, melanoma, mesenchymal, neuroendocrine, plasmacytoma, signet ring, squamous tumors). Altogether, these forms account for less than 2% of colorectal tumors. There are no specific diagnostic or therapeutic recommended approaches and most of the information available from literature derives from small and retrospective clinical series. In the present study, we provide a paramount and updated view on clinical and biologic characteristics of rare colorectal tumors.

## 1. Introduction

**Figure 1:**
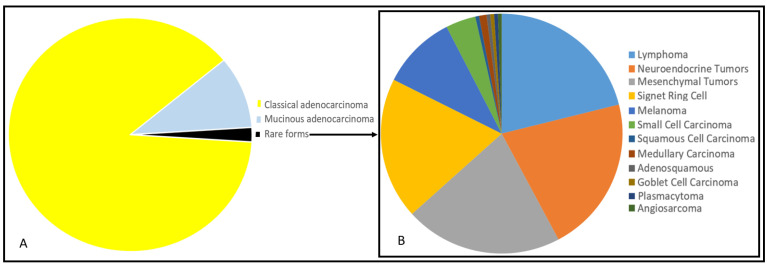
Representation of percentage of distribution of all colorectal cancers through pie chart (given a total frequency equal to 100) (**A**). Representation of percentage of distribution of rare forms of colorectal cancers (given a total frequency of rare forms equal to 100) (**B**). Different histotypes are indicated with colors in legends besides the pie charts. Data are extracted and elaborated from the American Cancer Society (Cancer Facts and Figures 2010. Atlanta: American Cancer Society; 2010).

**Figure 2:**
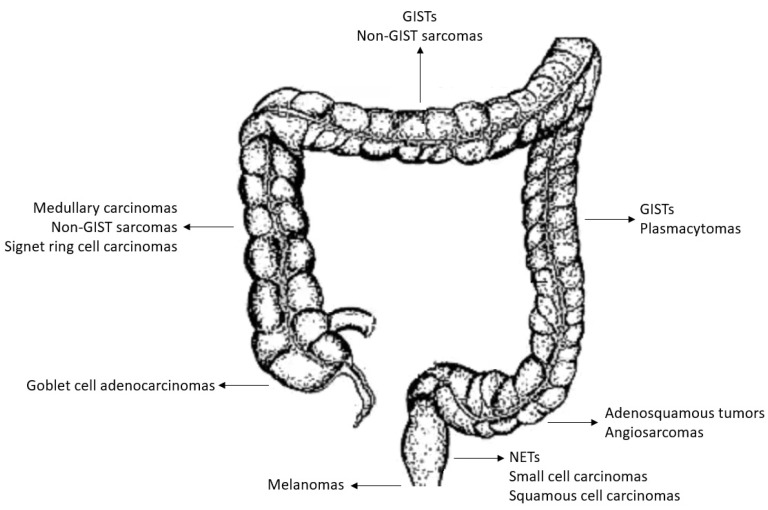
Anatomical site representation of predominant localization (as derived from the analysis of the clinical series analyzed in the present work).

Colorectal cancer (CRC) is the third leading cause of cancer-specific death. It has been estimated that, in 2018, there were 1.8 million new cases of CRC in Western countries. The most frequent form of CRC is represented by (classical and mucinous) adenocarcinoma with three grades of differentiation: well, moderate, poor [1]. Adenocarcinomas represent about 98% of tumor histological types (about 1,764,000 cases) being moderately differentiated form the most frequently documented in the pathology reports (about 80%). However, other rare histotypes can be found in colon and rectum (Figure 1) [2]. Anatomical site prevalence is reported in Figure 2. Altogether, even if probably underestimated (see beyond), these forms account for less than 2% of colorectal tumors. There are no specific diagnostic or therapeutic recommended approaches and most of the information available from literature derives from small and retrospective clinical series.

In the present study, we reviewed all the rare forms of CRCs so far described in literature with a potential diagnostic and clinical impact in the oncological practice in order to provide a paramount view on their clinical characteristics and to give an updated descriptive focus on their molecular characteristics.

## 2. Methods

We reviewed the published articles using PubMed and Scopus databases. The articles were selected and discussed among authors and the clinical information was enriched by genetic data (Table 1). The search was performed in August 2021 with the terms “colorectal tumors” OR “colon” OR “rectum” AND “rare tumor” OR “rare histotype”. No filters were applied (type of study or publication date). Nine-hundred and eighty articles were retrieved. Only studies reporting on malignant neoplasms were analyzed. Additionally, relevant reviews on specific topics were analyzed to find specific neoplastic forms and repeat the search by including in the search terms the eligible histotypes. Only studies written in English language were included. Mucinous adenocarcinoma, a distinct subtype of adenocarcinoma, was not included in this review because it cannot be considered a rare histotype being present in about 10% of CRC patients. The tumors were divided into two groups: >1% of CRCs (rare tumors of colon and rectum), <1% of CRCs (exceptional tumors of colon and rectum). Information about genetic characteristics of tumors has been given to enrich the clinical overview of rare colorectal cancers. However, some information has been derived mostly from case reports (due to the rarity of the diseases); the latter represents a limitation, and the reported genetic data must be intended as hypothesis-generating, warranting further research.

## 3. Rare Tumors of Colon and Rectum

### 3.1. Lymphomas

Less than 5% of gastro-intestinal lymphomas (accounting for 5–10% of all non-Hodgkin’s lymphomas) arise into the colon and rectum [3]. However, colorectal non-Hodgkin’s B cell lymphomas together with GISTs (gastro-intestinal stromal tumors) and well-differentiated neuroendocrine tumors are among the three commonest malignant neoplasms after adenocarcinoma. The appendix is the most frequently affected site (60–70%), followed by the right (about 20%) and the left colon (about 10%). The most frequent histological subtype (80–90%) is Diffuse large cell B lymphoma (DLBCL) mainly occurring between 50–60 years. Other forms are MALT (mucose-associated lymphoid tissue), Burkitt, enteropathy-associated T-cell, mantle cell, and follicular lymphoma [4]. The male:female ratio is about 1.5:1. No single marker is specific for lymphoma diagnosis and additional panels are applied on the basis of a first morphologic assessment. Most commonly used are T- and B-cell markers (CD3, CD5, CD20, and CD79a), and leukocyte common antigen (LCA). The clinical presentation is not different from the classical adenocarcinoma (“B symptoms” are frequently absent) being very heterogeneous and depending on the involved site. The treatment is based on surgery and chemotherapy, however, in many cases, when the first biopsy allows a correct diagnosis and obstruction and/or bleeding are not present, poly-chemotherapy and anti-CD20 immunotherapy can induce complete and long-lasting responses making the surgery unnecessary [5]. Stage at presentation is the most important prognostic factors for survival. *MYC* and *BCL2* genes are frequently altered in colorectal DLBCL. Notably, the co-expression of the two gene products identifies an aggressive DLBCL subtype [6]. Other cytogenetic abnormalities involving also other histological forms (i.e., follicular type) are translocations of *API2* (11q21), *BCL2* (18q21), *BCL6* (3q27), *IGH* (14q32), *IGK* (2p12), *IGL* (22q11), *MYC* (8q24), and *MALT1* (18q21) [7,8]. The prognostic value of these genetic alterations in colorectal lymphomas is debated and under investigation.

### 3.2. Mesenchymal Tumors

Mesenchymal neoplasms of colon and rectum represent about 1% of all CRCs and they are diagnosed predominantly as unexpected findings at histopathological examination after surgical removing of a primary intestinal tumor mass mimicking an adenocarcinoma [9,10]. Pre-surgical histopathologic, clinical, or radiologic diagnosis is extremely difficult. In fact, in most cases, a certain diagnosis is performed on a tumor sample from a large surgical resection. In the majority of reported cases, sarcomas of the colon and rectum present with non-specific symptoms.

#### 3.2.1. GISTs

GISTs are the most common sporadic mesenchymal tumor of the colon and rectum and about 10% of gastro-intestinal GISTs arise in this anatomical site. They are more common in the left and transvers colon and originate from Cajal cells that work as pacemaker of the smooth muscle. In fact, the large part of GISTs shows immunophenotypic similarities to Cajal cells with positive staining for CD117, CD34, and vimentin [10,11]. Histologically, not all GISTs are composed of spindle cells, which accounts for only 70% of GISTs, and other subtypes, such as epithelioid cells and mixed spindle and epithelioid cells, account for 20% and 10% of GISTs. Therefore, the molecular and genetic biomarkers provide additional information for the diagnosis of GISTs. The incidence is estimated to be about 10 cases per million per year in USA [11]. Generally, they present with a growing and bleeding trans-mural mass. Obstruction and abdominal pain are late events. Mean age of presentation is about 60 years [12]. In about 80% of cases, the neoplasm is sporadic and it is driven by activating mutations of type III receptor tyrosine kinase (c-*KIT* or CD117) in exons 11. Exon 9 mutations are more frequent in small bowel GISTs [13]. A proper molecular and histologic diagnosis of colorectal GIST is necessary since the introduction of therapies targeting the KIT proto-oncogene (imatinib and second-line tyrosine kinase inhibitors: sorafenib, dasatinib, and nilotinib) [14].

KIT alteration is the most common genetic change in GISTs. However, in recent years many other genes have been found altered including *PDGFRα* (platelet-derived growth factor receptor α) (35% of GISTs without *KIT* mutations) in exons 8, 12, 14, *BRAF* (B-raf proto-oncogene) V600E mutation, *NRAS, KRAS* [15,16]. Interestingly, *PDGFRα* mutated colorectal GISTs have similar response rate to *KIT*-targeted therapies with the exception of the D842V variant which is refractory to standard kinase inhibition [17]. *BRAF* V600E, *KRAS*, and *NRAS* mutated GISTs are rare (<5%), more aggressive and more frequent in small bowel. GIST with no mutations in the previously mentioned genes were considered wild-type; however, these can have a *SDH* (succinate dehydrogenase) gene mutation [18]. The latter is an enzyme that catalyzes the oxidation of succinate to fumarate. Deficiency in *SDH* produces a succinate accumulation, which, in turn, inhibits TET (Ten-Eleven Translocation) family of DNA hydroxylases. TET proteins catalyze the demethylation of 5-methylcytosine on DNA [19]. The final result is an alteration of DNA methylation status and thus of genes’ expression. SDH mutations have not been so far described in colorectal GISTs.

#### 3.2.2. Non-GIST Sarcomas

Most of the non-GIST sarcomas raising in colon and rectum are leiomyosarcomas and liposarcomas [20]. Notably, according to the World Health Organization Classification of Tumors of the soft tissue [21], liposarcoma can be classified in five subtypes: well-differentiated/atypical lipomatous tumor, myxoid, round cell, pleomorphic, and dedifferentiated. Age > 75 years and colon localization are bad survival prognosticators [22,23]. Most of these tumors involving the colon are well-differentiated or dedifferentiated forms, probably arising from the retroperitoneum. The prognosis is dismal because of a tendency to spread to distant organs. The treatment is based on systemic chemotherapy with doxorubicin and ifosfamide as first line with outcomes varying in different histotypes [24,25]. Other drugs—including dacarbazine, gemcitabine, and docetaxel—are widely used in subsequent treatment lines. Interestingly, *MDM2* amplification and *CDK4* polisomy have been observed in colorectal dedifferentiated liposarcomas [26]. Occurrence of MDM2 amplification can be studied by FISH (fluorescence in situ hybridization) or immunohistochemistry [27]. Other genetic features of well-differentiated liposarcomas are amplification of the 12q13–15 (including *MDM2, SAS, HMGA2*, and *CDK4* genes), 1q21–24, 6q22–24, 20q13, or 12q24 regions, deletions of 13q14–21 or 11q22–23, and *TP53* mutation [28].

Schwannomas represent about 5% of all mesenchymal tumors and derive from the Schwann cells producing the myelin sheaths of peripheral nerves. The predominant origin in the gastro-intestinal tract is from Auerbach’s myenteric plexus [29]. Unfortunately, due to the very small number of cases reported in literature, incidence rates and clinical characteristics (age, gender, primary tumor site, lymph nodes involvement, association with von Recklinghausen disease, recurrence rates, etc.) of colorectal schwannomas cannot be defined. In fact, more than 80% of gastrointestinal schwannomas arise into the stomach [30]. Furthermore, nor clinical/endoscopic presentation neither radiologic imaging findings are specific. However, a submucosal lesion at endoscopy with a well-defined homogenous mural nodule with low enhancement at computed tomography (CT) scan can suggest a schawannoma [31]. Pathological examination, including immunohistochemical analysis of a biopsy performed by colonoscopy, is the definitive diagnostic approach. Usually, these tumors appear as spindle cell aggregates, showing positivity for S-100, vimentin, and glial fibrillary acidic protein; and negativity for CD117, CD34, actin, or cytokeratins. The differential diagnosis includes other mesenchymal and neuro-ectodermal tumors. However, KI-67 staining rarely exceeds the 3% [32]. Total-body CT scan is the gold-standard for both pre-operative staging and follow-up of colorectal schawannomas. Surgery with radical resection is the mainstay treatment. The use of adjuvant/post-surgery interventions, including radiotherapy and/or adjuvant chemotherapy, cannot be recommended due to the absence of specific clinical studies. The prognosis is good because of the predominant benign nature of the tumor. Lymph-nodal involvement and/or hepatic recurrences are observed in very rare colorectal ‘malignant’ schwannomas (better termed as malignant peripheral nerve sheath tumor) [33]. Notably, colorectal schwannomas are sporadic, not associated with neurofibromatosis I or II (NF1 and NF2). Furthermore, they do not present *NF1* and *NF2* genes alterations although loss of *NF2* occurs in 60% of sporadic extra-colorectal schwannomas [34,35]. Unfortunately, studies on genomic landscapes of colorectal schwannomas are not reported due to the rarity of the disease and the greater attention paid to cranial and spinal cases.

### 3.3. Neuroendocrine Tumors

Rectal and colon well-differentiated neuroendocrine tumors (NETs), “ex carcinoids”, are the predominant form of colorectal NETs and about 30% and 5% of all gastrointestinal NETs, respectively [36]. More than 50% of them are asymptomatic. Proliferation ratio and presence of necrosis are two features used to distinguish the grade of differentiation of NETs (well-differentiated NETs vs. carcinomas) [37,38]. Notably, they are not to be confused with goblet cell adenocarcinomas (see beyond) rare tumors occurring in appendix that are different from typical well-differentiated neuroendocrine tumors showing both gland and neuroendocrine differentiation: these tumors are treated as conventional adenocarcinomas. Typical biomarkers of the neuroendocrine phenotype are chromogranin A (CgA), synaptophysin, and CD56—often used for diagnostic distinction from non-neuroendocrine neoplasms.

They rarely induce hormones syndrome (flushing, diarrhea, hypotension, etc.) and are often discovered incidentally during routine endoscopic examinations or surgery for acute inflammation [39]. Some symptoms are overlapping with those of primary adenocarcinomas: rectal bleeding, pain, and change in bowel habits. Obstruction is not frequent. Survival for rectal well-differentiated neuroendocrine tumors encompasses 85% at five years while it is about 60% for colon well-differentiated NETs [40]. In fact, the large part of colorectal NETs has a benign behaviour. In less than 20% of cases, although well-differentiated, some NETs have a malignant aggressive phenotype. The risk of malignant behaviour depends on tumor size (generally larger in colon than rectal well-differentiated NETs) and depth of invasion. In fact, rectal NETs larger than 2 cm invading the muscularis propria are associated with distant metastases and poor prognosis [41].

The male:female ratio is about 1.1:1 and the mean age at diagnosis about 60 years. After diagnosis, obtained through pancolonscopy and/or endoscopic ultrasonography (particularly in rectal NETs), colorectal NETs are managed as those arising in other tissues [42]. In particular, standard contrasted total-body CT or MRI (magnetic resonance imaging) of the abdomen and pelvis are done for staging the disease. Octreoscan is used for the evaluation of somatostatin receptors (SST2) by metastases allowing therapeutic use of octreotide analogues. Analysis of 5-hydroxyindoleacetic acid (5-HIAA) is not recommended in colorectal NETs, since a very small fraction secretes serotonin or hormones [43]. CgA, with limitations discussed elsewhere, can be monitored in patients with metastatic disease or after radical resection of tumor [44].

Rectal well-differentiated NETs smaller than 2 cm can be treated with endoscopic resection considering their very low metastatic potential and the tendency to be confined in the mucosa/submucosa. Tumors larger than 2 cm or those localized in other sites of the colon need to be treated with surgical excision similarly to adenocarcinomas [45]. The medical treatment of poly-metastatic disease overlaps with that of other gastrointestinal high-grade NETs [42]. The latter, also called neuroendocrine carcinoma, occurs very rarely in colon and rectum compared to well-differentiated NETs, they include small-cell and large-cell neuroendocrine carcinomas. Interestingly, mixed variants with neuroendocrine, adenocarcinoma or squamous cells are also described. Compared to well differentiated forms their biologic and clinical behaviour is highly aggressive. Lymph-nodes and/or liver metastases are present in more than 80% of cases [46]. Cisplatin-based chemotherapy remains the mainstay of first-line treatment in advanced disease. However, in recent years, the therapeutic scenario of these forms has expanded considerably with new systemic therapies including somatostatin analogs (SSAs) and peptide receptor radionuclide therapy (PRRT) in patients expressing SS-receptors, mTOR, and tyrosine kinase inhibitor (TKI) inhibitors for patients progressing to both previous chemotherapy and new chemotherapy schedules (temozolomide and capecitabine) [47].

There are very limited data about key-driver mutations in colorectal NETs. Interestingly, in a study reporting targeted pyrosequencing of *BRAF, RAS*, and *PIK3CA*, no mutations were found in colorectal well-differentiated NETs suggesting that neither ERK nor AKT downstream pathways are crucial in tumorigenesis of these tumors [48]. Mutational landscape assessed through next-generation sequencing (NGS) according to a restricted panel of 50 genes revealed *FBXW7, TP53, MET, K-* and *N-RAS, ERBB4, RB-1, PTEN, SMAD4, EGFR, ATM, CDKN2A*, and *KIT*, as commonly mutated genes in appendiceal and rectal well-differentiated NETs. A lower mutation rate was also described in *SMARCB1, IDH1, ALK, VHL, AKT1, RET, STK11, FLT3, BRAF, CTNNB1, ERBB2, EZH2, HNF1A*, and *SMO*. Repetitive mutations (more than one case) in *TP53* [p.R337C and p.R213 * (*: stop-gain variant)], *PTEN* (p.W111 *, p.Q214 *), *CDKN2A* (p.W110 *), *FBXW7* (p.R465H), and *AKT1* (p.R23Q) were found in rectal well-differentiated NETs but not in appendiceal ones. No repetitive mutations were described in appendiceal well-differentiated NETs. Interestingly, *PTEN* (p.G129R), *EGFR* (p.E709K), and *KIT* (p.V555I) were shared between rectal and appendiceal NETs [49]. Furthermore, in contrast to adeno- or neuroendocrine carcinomas, NETs do not exhibit microsatellite instability.

## 4. Exceptional Tumors of Colon and Rectum

### 4.1. Adenosquamous

There are no specific registries for these exceptional forms of colorectal tumors and it has been estimated that less than 80 new cases/year occur in the United States (0.025% of all colorectal neoplasms) [50]. Most of them arise in the sigmoid colon. Associations with ulcerative colitis, schistosomiasis, polyposis, and other cancers, have been reported [51]. The clinical presentation is similar to the classical adenocarcinoma. Histological examination reveals mixed aspects of glandular and squamous epithelial differentiation with the glandular areas with positive staining for carcinoembryonic antigen (CEA) and CK7 [52]. However, serum CEA level is generally normal. Metastases can occur to the liver, lymph-nodes, lung, and peritoneum. Interestingly, a paraneoplastic syndrome of hypercalcemia is frequently revealed [53]. Since squamous cells are not present in colorectal mucosa, some theories have been proposed: (1) degeneration of ectopic ectodermal cells; (2) evolution towards a squamous metaplasia of the mucosa; or (3) cancerous transformation of pluripotent stem cells with high differentiation plasticity [51,52]. The gold standard of treatment is surgery. The role of adjuvant therapy is completely undefined. The prognosis is uncertain and it depends mainly on the presence of metastases. In general, patients affected by adenosquamous carcinoma have a lower survival compared to adenocarcinoma patients with the same stages of disease [52]. Genomic landscapes of these histotypes are unknown.

### 4.2. Angiosarcoma

Angiosarcoma accounts for less than 1% of all colorectal sarcomas and it is extremely rare [54]. Only a few cases have been reported in literature and their genetics is not known. The clinical presentation is not specific as well as the diagnostic and staging work-up. The diagnosis is done at post-surgical histologic examination. Tumor areas are rich in biphasic spindle and epithelioid cells with vasoformative tendency; positive staining for endothelial markers (CD31, CD34, factor VIII, and FLI-1), is diagnostically relevant. Radical excision with tumor-free wide margins is mandatory. Loco-regional lymph-nodes involvement is infrequent (2–12%) and it is associated with poor prognosis along with visceral localization and residual disease [55,56]. No adjuvant chemo or radiotherapies are known. The advanced phase of the disease is treated according to standard systemic therapies in soft tissue sarcomas [24,25]. Due to the rarity of the disease, there are no specific genetic studies exploring any specific mutational signatures.

### 4.3. Goblet Cell Adenocarcinomas

Goblet cell adenocarcinoma (GCC) is a subtype of appendiceal cancer. It is a tumor composed by neuroendocrine and epithelial cancerous cells with positive staining for both glandular and neuroendocrine differentiation markers (CEA, synaptophysin, and CD56). The incidence is about 1 per 2 million individuals/year and it affects mainly patients 50–60 years old [57]. The clinical presentation is not specific usually mimicking appendicitis. The diffusion occurs predominantly into the abdomen (carcinomatosis). The correct diagnosis is done at histologic examination after appendectomy for appendicitis, or surgery for an intestinal blockage due to one or multiple growing masses. In women, GCC frequently involves the ovary, making the differential diagnosis with ovarian cancer challenging [58]. The relative abundance of the two components (neuroendocrine vs. adenocarcinoma) influences both prognosis and treatment [57,58]. In fact, the prognosis worsens as the adenocarcinoma component increases. Surgical excision of the tumor with cancer-free margins is the mainstay of the treatment. The standard systemic work-up is similar to that of colon cancer. In case of peritoneal carcinomatosis, cytoreductive surgery followed by HIPEC (hyperthermic chemotherapy) may be considered [59]. Interestingly, the genomic landscape of GCCs is quite distinct from adenocarcinomas and NETs. *TP53* and *BRAF* (p.V600E) can be altered in GCC. Furthermore, *CTNNA1, NOTCH1, NUMA1, TGFBR2, USP9X*, and *ERBB2* mutations have been described. Alterations in ‘typical’ adenocarcinoma key-driver genes—including *APC, KRAS, NRAS, PIK3CA, PTEN,* or *SMAD4* —have not been detected so far [60].

### 4.4. Medullary Carcinoma

Medullary carcinoma of the colon is an extremely rare tumor representing 0.05% of all colon cancers [61]. Clinical and radiologic characteristics are not specific mimicking classical adenocarcinoma. The mean age at diagnosis is about 70 years with a female:male ratio of 2:1. The proximal colon is the most common large intestine localization [62]. Metastases development is rare also in cancers reaching large bulky masses. On a morphologic point of view, it presents a homogeneous solid growth pattern of non-glandular poorly differentiated malignant cells. Despite these neuroendocrine features, the cells show positive staining for TFF-2, MUC-1, and MUC-2. Microsatellite instability and CD8+ lymphocytes infiltration are frequent [63]. Other peculiar biologic characteristics are diploidy, loss of *MLH-1* and *CDX2* and strong expression of calretinin [64]. Studies through CGA (Cancer Genome Atlas) data analysis revealed upregulation of genes induced by IFNγ: *IDO-1, WARS, GBP1, GBP4, GBP5, PD-1*, and *PD-L1*. Mutations have been described in *ARID1A* (p.E896*) determining loss of protein function and expression, and *BRAF* (p.V600E) genes [65].

### 4.5. Melanomas

Melanoma can arise in extra-cutaneous tissues including uvea, leptomeninges, and mucous membranes. About 1% of melanomas can present in the gastrointestinal tract. The most frequent localization is in the anorectal region (more than 50% of cases), while the remaining large intestine is involved in less than 1% of cases [66]. About 60% occurs in the anal canal, up to 40% in the rectum. Clinical presentation is not specific overlapping with that one of classical adenocarcinomas. A typical rapidly growing grey–brown lesion is often seen in anal localizations. Standard immuno-histochemical staining shows classical positivity to S100 protein, Melan-A, HMB-45, and vimentin, and negativity for CK-7, SMA, LCA, CD117, AE1/AE3, and CD138 [67]. Since melanocytes are not evolutionarily present in such internal organs (being that they are not exposed to the sun), it is worth discussing the cell origin of these tumors. A first explanation of colorectal (non-anal) melanomas could be based on the occurrence of metastatic lesions after spontaneous regression or occult synchronous cutaneous primary melanoma (it is estimated about 20% of cases). Histological examination does not always help to differentiate these forms. Other theories consider the very rare presence of melanocytes into the colon from neural crest cells (“ectodermal differentiation theory”) or from anal melanocytes or from multipotent stem cells [68,69]. However, clinician should ever verify: (1) a thorough dermatological screening including inspection of eyes, anus and vagina; (2) eventual previous surgical resections of skin tumors; (3) history of ocular melanoma. In localized disease, the mainstay of treatment is the surgical removal of primary colorectal melanoma. After radical resection and definition of pathologic staging, there are no standard adjuvant therapeutic strategies particularly when loco-regional lymph-nodes are involved [67]. Advanced disease is treated in the same way as the non-colorectal melanomas. The prognosis of colorectal localization of melanoma is dismal because locoregional lymphnodal and distant metastases are frequently present at diagnosis [70]. Interestingly, *BRAF* and *NRAS* mutations are less frequent compared to cutaneous melanomas. By contrast, activating *KIT* mutations are reported in more than 30% of cases [71]. In these patients, imatinib has shown promising activity [72]. Other mutations in anorectal melanomas have been identified in *BRCA1, HRAS, MLH1, NF1, PDGFRA*, and *SF3B1* [73]. Some of these genes (*BRCA1* and *MLH-1*) are associated with genome instability and can be predictive of response to immunotherapy; others (*NF1*, *PDGFRα*, and *SF3B1*) are under intensive investigation for their potential ‘actionability’.

### 4.6. Plasmacytoma

Colonic solitary plasmacytoma is extremely rare (less than 1/10,000,000) with very few descriptions available in literature [74]. General clinic-pathological characteristics cannot be defined. The tumor involves colonic plasma cells (monoclonal proliferation) without evidence of bone marrow localization. The clinical and radiologic presentation are not specific. Colon biopsies often reveal undifferentiated cells. Serum protein electrophoresis and antibody components search in urine are generally normal [75,76]. Surgical resection and histological examination allow both treatment and diagnosis, showing kappa light chain+, CD38+, CD3−, CD20−, CD43− plasmacytoid cells [77,78]. Neither neo-adjuvant nor adjuvant evidence-based chemo- or radiotherapies can be applied. The treatment of advanced disease is similar to that one of multiple myeloma. The prognosis is good with five-year survival of about 90% in localized disease and no lymph node involvement [75]. Data on genomic landscape of colon plasmacytoma are not available.

### 4.7. Signet Ring Cell Carcinoma

Signet ring cell carcinoma (SRCC) represents about 1% of CRCs. It has an intracytoplasmic mucinous component that pushes the nuclei to the periphery of cell, conferring it the typical appearance of a “signet ring”. SRCC of the colon frequently expresses MUC2, CDX2 (nuclear staining), and MUC5AC [79]. The mean age at diagnosis is 50 years, the male:female ratio 1:3. With the exception of a constant non-polypoid morphology, clinical and radiologic findings are indistinguishable from the adenocarcinoma. However, it has a poor prognosis compared to classical and mucinous adenocarcinomas and a strong association with inflammatory bowel diseases has been described [80]. Loco-regional lymph node metastases and peritoneal dissemination are frequent. Five-year survival is less than 5% [81]. Study through NGS restricted gene panels showed that incidence of *K-* and *N-RAS*, *PIK3CA, APC*, and *TP53* mutations are lower compared to adenocarcinoma suggesting that alternate pathways may be involved in SRCC tumorigenesis [82].

### 4.8. Small Cell Carcinomas

Primary small cell carcinoma or small cell undifferentiated carcinoma or small cell neuroendocrine carcinoma of the colon is an aggressive tumor, showing early locoregional and distant metastases. It is a subtype of neuroendocrine carcinomas. It is treated separately from other neuroendocrine tumors due to its exceptional incidence. In fact, these tumors account for about 0.2% of all CRCs (the most frequent gastro-intestinal site is the esophagus) [83,84]. An adenocarcinoma component is rarely present and associated with better prognosis. As in the typical pulmonary localization, histological examination shows solid clusters of small sized cells with very frequent lymphovascular invasion and positivity to synaptophysin [85]. There are no prospective studies on the specific therapeutic management. The treatment is restricted to empiric and pragmatic considerations and similar to that one used in small cell lung cancer of the lung due to the absence of specific studies [86]. The two-year survival is less than 20%. Restricted genes analyses showed a rare mutation of *TP53* (p.Q331 *) and no alterations of *KRAS* [87].

### 4.9. Squamous Cell Carcinoma

Squamous cell carcinoma arising in colon and rectum is a very rare tumor. The incidence is estimated to be less than 1 on 100,000 patients affected by a colorectal tumor [88]. The etiology is unknown. The diagnosis is primarily based on the morphologic appearance of the tumor rather than on marker assessment. Some theories are based on unclear influences of papilloma viruses, oncogenic aberrant differentiation of basal stem cells, and/or squamous metaplasia [89,90]. The treatment of localized disease relies on surgical excision of the primary tumor with cancer-free margins followed by adjuvant chemo- and radio-therapy (i.e., combination of 5-fluorouracile + mitomycin-C and radiotherapy at 45 Gy) at least in T3 and T4 tumors (N0/N+) [91]. A pre-operative treatment similar to that of anal squamous cell carcinoma can be adopted in rectal localizations. The prognosis compared to the same stages of classical adenocarcinoma is worse [92]. Systemic treatment of advanced disease is based on the same drugs used in anal squamous cell carcinoma. Data on genomic landscape of colorectal squamous carcinomas are lacking.

## 5. Discussion

We have provided a descriptive overview of rare forms of CRCs. However, some issues deserve to be raised and discussed.

First, specific staging systems for these forms of CRCs are currently lacking due to their rarity. Thus, the AJCC (American Joint Committee on Cancer) TNM staging system working for the classical adenocarcinoma is applied. However, it does not take into account some biological characteristics (size, mitotic index, mutations, etc.) that could have a major role in some forms. This suggests the need for including in the staging systems molecular or biological data with both pathogenic and therapeutic significance. Moreover, the prognostic value of some characteristics overcome that of the volume of masses (in an eventual classical T assessment) such as necrosis in GISTs, density or anarchy of vascular channels in angiosarcomas, squamous components in mixed forms, etc.

Second, the improvement of the understanding in biology and pathogenesis of these forms is required and it could inform about the clinical significance of specific mutations (unique for some of them) in publicly available archives (i.e., ClinVar, CancerMine, OncoScore, CIViC, etc.). These tools provide practical information for interpreting the clinical and diagnostic significance of some variants and may also help researchers to drive and focus their scientific efforts.

In our opinion, improvement of diagnostic tools, including molecular assessment with NGS, will allow better definition of the histologic entities of CRCs. Consequently, it is argued that, in this case, we will be able to increase the diagnosis of ‘rare’ forms of CRCs. This is a phenomenon recently described for NETs and thyroid cancer: in synthesis, the amelioration of detection tools (more sensitive imaging and laboratory methods) optimizes the diagnosis inducing an incidence increase [93,94]. A similar trend could be revealed in the next future particularly in the mixed histotypes of CRCs (adenocarcinoma plus other cellular types). In fact, in this context, NGS can reveal specific genetic features confused by apparently similar morphological characteristics. This ‘diagnostic revolution’ will help pathologists to improve and refine their diagnostic power.

Furthermore, CRC cases are classified according to standardized codes from the International Classification of Diseases (ICD). The code for oncology integrating topographical, morphological and biologic information for CRC is ICDO3-M 8140.3. The question has an apparent formal or bureaucratic significance, but it deserves appropriate discussion. In fact, although specific morphological codes exist for rare entities, they are often not documented in the pathology reports. In other words, assignment of generic morphological codes for CRC accounts for an underestimation of rare CRC frequency into National Tumor Registries as well as of related financial needs. Therefore, awareness of pathologists about the importance to register by using precise codes should be improved.

The present study has at least two limits. First, the paucity of specific data on genetic alterations do not give the chance to draw any conclusion on the involvement of the genes in the biology and pathogenesis of the tumors. Second, the source of data is limited by the intrinsic rarity of these tumors as stated in Methods. Thus, the role of many clinic-pathological and prognostic characteristics deserves confirmation in larger series.

## 6. Conclusions

The lack of specific molecular or biologic signatures can make differentiation between different histotypes of this group of tumors difficult and lead to misinterpretations based only on a ‘morphologic’ point of view. In this context, the emergence of NGS techniques will open a new scenario based upon the complete exome sequencing of these rare tumors to find somatic mutations that could have diagnostics and therapeutic consequences. In fact, day by day, the therapeutic weaponry available for the treatment of tumors is increasing giving the opportunity to include the patients in ‘basket’ trials for the evaluation of the efficacy of these agents in these orphan-drug diseases. Hopefully, implementation of specific registries to collect, systematize, and connect clinical, therapeutic, and genetic information beyond the difficult and utopian design of prospective clinical trials is warranted.

## Figures and Tables

**Table 1:** Clinical, epidemiologic, and genetic information according to rare histotypes of colorectal cancer.

Histotype	Incidence *	Main Negative Pathologic Prognostic Factors	Overall 5-Year Survival in Advanced Disease	Described Association with Inflammatory Bowel Diseases?	Putative Altered Genes
Lymphoma	1/50,000	Involvement of loco-regional lymph-nodes co-mutations of MYC and BCL2	40%	Yes	*MYC, BCL2*, translocations: *API2* (11q21), *BCL2* (18q21), *BCL6* (3q27), *IGH* (14q32), *IGK* (2p12), *IGL* (22q11), *MYC* (8q24), and *MALT1* (18q21)
GIST (Gastro-Intestinal Stromal Tumor)	1/100,000	Size > 2 cm high mitotic index	50%	No	*BRAF, KIT, PDGFRA, K- N-RAS, SDH*
Well-differentiated neuroendocrine tumor	1/100,000	Size > 2 cm high mitotic index	15%	No	*ATM, CDKN2A, EGFR, ERBB4, FBXW7, KIT, MET, PTEN, K- N-RAS, RB-1, SMAD4, TP53* (commonly mutated genes). Others: *AKT1, ALK, BRAF, CTNNB1, ERBB2, EZH2, FLT3, HNF1A, IDH1, RET, SMARCB1, SMO, STK11, VHL*
Signet ring cell	1/125,000	Involvement of loco-regional lymph-nodes	<5%	Yes	*APC, PIK3CA, K- N- RAS, TP53* (lower compared to adenocarcinoma)
Small cell carcinoma	1/250,000	Involvement of loco-regional lymph-nodes	5%	Yes	*TP53*
Adenosquamous carcinoma	1/375,000	Involvement of loco-regional lymph-nodes High percent of squamous cell component	10%	Yes	Unknown
Non-GIST mesenchymal tumor	1/500,000	High mitotic index	5%	No	Amplifications of *CDK4, HMGA2, MDM2,* *SAS*, of 1q21–24, 6q22–24, 20q13, 12q24 regions, deletions of 13q14–21 or 11q22–23. *TP53*
Medullary carcinoma	1/3,000,000	Involvement of loco-regional lymph-nodes	Not definable ***	Yes	*ARID1A, BRAF, CDX2, GBP1, GBP4, GBP5, IDO1, MLH1, PD-1, PD-L1, WARS*
Goblet cell adenocarcinoma	1/10,000,000	High mitotic index	18%	No	*BRAF, CTNNA1, ERBB2, NOTCH1, NUMA1, TGFBR2, TP53, USP9X*
Angiosarcoma	Unknown **	Involvement of loco-regional lymph-nodes	10%	No	Unknown
Melanoma	Unknown **	Involvement of loco-regional lymph-nodes	5%	No	*BRAF, NRAS* (mutations are less frequent compared to cutaneous melanomas). *BRCA1, HRAS, KIT, MLH1, NF1, PDGFRA, SF3B1*
Plasmacytoma	Unknown **	Involvement of loco-regional lymph-nodes High mitotic index	70%	No	Unknown
Squamous cell carcinoma	Unknown **	Involvement of loco-regional lymph-nodes	30%	Yes	Unknown

* 1 case/population/year. Data are elaborated and normalized from data of the American Cancer Society (Cancer Facts and Figures 2010. Atlanta: American Cancer Society; 2010.) in the USA population in 2010. ** Reasonably < 1/10,000,000. *** Not definable from data available in scientific literature.

## Data Availability

Data sharing not applicable.
